# Chemical Composition, Antibacterial and Antibiofilm Actions of Oregano (*Origanum vulgare* subsp. *hirtum*) Essential Oil against *Salmonella* Typhimurium and *Listeria monocytogenes*

**DOI:** 10.3390/foods12152893

**Published:** 2023-07-29

**Authors:** Sonia Kolypetri, Dimitra Kostoglou, Anastasios Nikolaou, Yiannis Kourkoutas, Efstathios Giaouris

**Affiliations:** 1 Laboratory of Food Microbiology and Hygiene, Department of Food Science and Nutrition, School of the Environment, University of the Aegean, 81400 Myrina, Lemnos, Greece; 2 Laboratory of Applied Microbiology and Biotechnology, Department of Molecular Biology and Genetics, School of Health Sciences, Democritus University of Thrace, 68100 Alexandroupolis, Greece

**Keywords:** oregano essential oil, foodborne pathogenic bacteria, minimum inhibitory and bactericidal concentrations, biofilms, minimum biofilm eradication concentration, GC-MS, gene expression, RT-qPCR, virulence, food safety

## Abstract

Essential oils (EOs) are plant mixtures that are known to present strong bioactivities, including a wide antimicrobial action. Biofilms are microbial sessile structures that represent the default mode of growth of microorganisms in most environments. This study focused on the antimicrobial action of the EO extracted from one of the most representative oregano species, that is, *Origanum vulgare* (subsp. *hirtum*), against two important foodborne pathogens, *Salmonella enterica* (serovar Typhimurium) and *Listeria monocytogenes*. For this, the minimum inhibitory concentrations of the EO against the planktonic and biofilm growth of each bacterium were determined (MICs, MBICs), together with the minimum bactericidal and biofilm eradication concentrations (MBCs, MBECs). The EO was also analyzed for its chemical composition by gas chromatography—mass spectrometry analysis (GC-MS). The influence of EO exposure on the expression of some important virulence genes (*hly*, *inlA*, *inlB* and *prfA*) was also studied in *L. monocytogenes*. Results revealed a strong antibacterial and antibiofilm action with MICs and MBICs ranging from 0.03% to 0.06% (*v*/*v*) and from 0.06% to 0.13% (*v*/*v*), respectively. The application of the EO at 6.25% (*v*/*v*) for 15 min resulted in the total eradication of the biofilm cells of both pathogens. The EO was mainly composed of thymol, *p*-cymene, *γ*-terpinene and carvacrol. The 3 h exposure of *L. monocytogenes* planktonic cells to the EO at its MBIC (0.06% *v*/*v*) resulted in the significant downregulation of all the studied genes (*p* < 0.05). To sum, the results obtained advocate for the further exploitation of the antimicrobial action of oregano EO in food and health applications.

## 1. Introduction

The genus *Origanum* L. comprises several species which differ in morphology and chemical characteristics, with most of them being originated in the Mediterranean region [1]. One of the most notable species is *Origanum vulgare* subsp. *hirtum*, which is a widely used aromatic plant in foods, feeds, cosmetics, and daily care products, especially due to its content in essential oil (EO), mainly rich in carvacrol and thymol [2]. An increasing number of studies have examined various bioactivities of *O. vulgare* EO, including strong antimicrobial action against various bacteria and fungi [3]. Such bioactivities may, however, differ between the EO of the same species due to differences in chemical composition which in turn are due to parameters such as the soil and climate conditions of plant growth, cultivation method, harvesting period, anatomical part of the plant used for extraction and method used to extract the oil [4].

Biofilms are microbial sessile (surface-attached) structures that represent the dominant mode of growth of almost all microorganisms in most environments, both natural and man-made [5]. Biofilm formation by bacterial pathogens is a worrying ability due to the increased tolerance and resistance of the enclosed cells to antimicrobials and other physicochemical stresses [6]. Such recalcitrance of biofilm cells is the result of many factors that act in concert and are related to biofilm growth, such as the limited penetration and/or the inactivation of some antimicrobials through the extracellular biofilm matrix, the reduced growth rate, the altered gene expression and physiology of biofilm cells, together with the increased presence of dormant and super-durable cells called persisters [7]. Alarmingly, biofilm formation is considered a key virulence factor for a wide range of microorganisms that cause human infections [8].

*Salmonella enterica* and *Listeria monocytogenes* are two well-known pathogenic bacteria that are responsible for foodborne infections [9]. Based on the latest epidemiological data for Europe, in 2021, salmonellosis remained the second most common foodborne zoonosis in humans after campylobacteriosis. That year, *Salmonella* provoked 773 foodborne outbreaks (FBOs), which is the largest number of FBOs recorded for 2021 [10]. As in previous years, the three most commonly reported *Salmonella* serovars were *S*. Enteritidis (54.6%), *S*. Typhimurium (11.4%) and monophasic *S*. Typhimurium (1,4,(5),12:i:-) (8.8%), representing 74.8% of the 50,817 confirmed human cases. On the other hand, listeriosis concerned 2183 confirmed human cases, provoked 196 deaths, and resulted in a case fatality ratio of 13.7% which is the highest compared to all the other zoonoses monitored. Both *S. enterica* and *L. monocytogenes* can form robust biofilms on various surfaces encountered within the food production chain and in this way persist and contaminate foods [11,12]. Thus, the ability of these bacteria to form biofilms and/or be included in those formed by other species (e.g., those of spoilage microflora) is considered a strong contributing factor to their prevalence in foods and their virulence potential [13,14,15].

The increased tolerance and resistance of several pathogenic bacteria to antibiotics and/or disinfectants has led to the continuous search for novel antimicrobial agents [16,17,18]. Plants may be a promising source for such compounds mainly due to their great biological diversity, abundance, and safe and ecofriendly nature [19,20]. In the past few years, there have been numerous studies published on the antimicrobial and antibiofilm actions of various EOs including those of oregano species [21,22,23,24]. Given that the EOs have multiple targets against microbial cells (e.g., rupture of the cell membrane, coagulation of the cytoplasm, interference with DNA transcription, protein synthesis and enzymatic activity, disturbance of the proton motive force etc.), it is believed that this lowers the possibility for microorganisms to develop resistance [1]. In this study, we evaluated the antimicrobial action of the EO extracted from plants of *O. vulgare* subsp. *hirtum*, organically cultured on Lemnos Island (north-eastern Greece), against both planktonic and biofilm cells of *S.* Typhimurium and *L. monocytogenes*. The EO was also analyzed for its chemical composition by gas chromatography—mass spectrometry analysis (GC-MS). The influence of EO sublethal exposure on the expression of some important virulence genes (*hly*, *inlA*, *inlB*, and *prfA*) was also studied in planktonic *L. monocytogenes* cells through reverse transcription quantitative polymerase chain reaction (RT-qPCR).

## 2. Materials and Methods

### 2.1. Bacterial Strains and Preparation of Their Working Cultures

The bacterial strains that were used in this study were *S. enterica* str. FMCC_B137, which was kindly provided by Prof. George-John Nychas (Agricultural University of Athens, Athens, Greece), and *L. monocytogenes* str. AAL 20107, which was kindly supplied by Dr. Nikolaos Andritsos (Athens Analysis Laboratories S.A., Microbiology Laboratory, Metamorfosi, Greece). The strain of *S. enterica* belongs to serovar Typhimurium; it is an epidemic strain of phage type DT193 and was originally isolated from a human salmonellosis outbreak [25]. The strain of *L. monocytogenes* belongs to serovar 1/2b and was originally isolated from a mixed green salad [26]. Before use, both strains were maintained frozen at −80 °C in BHI broth (Lab M, Heywood, Lancashire, UK) containing 15% *v*/*v* glycerol. When needed, each strain was streaked on Tryptone Soya Agar (TSA; Oxoid, Thermo Fisher Specialty Diagnostics Ltd., Hampshire, UK) and incubated at 37 °C for 24 h (preculture). Working cultures were prepared by inoculating a single colony from each preculture into 10 mL of fresh TSB (Condalab, Torrejón de Ardoz, Madrid, Spain) and then incubating at 37 °C for 18 h. The final concentration of each working culture was ca. 10^9^ CFU/mL and its purity was always confirmed by streaking on TSA and incubating it at 37 °C for 24 h.

### 2.2. O. vulgare EO

The EO that was used in this work was extracted from dried plant material by hydro-distillation procedure as previously described [27] and was kindly donated by Dr. Nikolaos Paterakis, owner of the essential oil production and trading company Aegean Organics (Agios Dimitrios, Lemnos, Greece). The year of harvest was 2022. Besides oregano, this company produces EOs and decoctions of various other aromatic plants that are all grown under a certified organic culture. Upon receipt, the EO was kept in a dark glass vial in the refrigerator (4 °C).

### 2.3. Determination of Minimum Inhibitory and Bactericidal Concentrations of EO against Planktonic Bacteria (MICs, MBCs)

The MIC of the EO against the planktonic growth of each bacterial strain was determined following the broth microdilution method as previously described [27]. In brief, ten different concentrations were tested for the EO which were prepared by successive two-fold dilutions in TSB and ranged from 0.5 to 0.001% *v*/*v* (a stock solution of the EO at 1% *v*/*v* was initially prepared in TSB also containing 9% *v*/*v* ethanol). Then, 360 μL of each of those dilutions were placed in duplicate in the wells of a sterile 100-well Bioscreen honeycomb plate (Oy Growth Curves Ab Ltd., Turku, Finland), and inoculated with the tested bacteria so as to have an initial concentration of ca. 10^4−5^ CFU/mL. The plate was incubated at 37 °C for 24 h in the Bioscreen Co Pro instrument, which was adjusted to record the absorbance of the contents of each well every 30 min at 600 nm (A_600nm_). Pure sterile growth medium (TSB) was used as a negative control (NC). For each bacterial strain, the MIC of the EO was calculated as its lowest concentration, resulting in no increase in absorbance with respect to the NC. To determine the MBC, 10 μL of broth cultures were aspirated from all the non-growth wells of the MIC assay and spotted (in duplicate) on TSA plates, which were then incubated at 37 °C for 24 h. For each bacterial strain, the MBC of the EO was determined as its lowest concentration that reduced the initial inoculum by more than 99.9% (no appearance of colonies). Each experiment was repeated three times starting from independent bacterial cultures.

### 2.4. Determination of Minimum Biofilm Inhibitory Concentrations (MBICs)

The MBIC of the EO against the biofilm growth of each bacterial strain was determined following the crystal violet (CV) staining method as previously described [27]. In brief, eight different concentrations were tested for the EO which were prepared by successive two-fold dilutions and ranged from 0.25 to 0.002% *v*/*v*. These dilutions were done in the respective growth medium for each strain (see below). Each bacterium was left to form biofilm on 96-well polystyrene (PS) microtiter plates (transparent, flat, Cat. No. 30096, SPL Life Sciences, Gyeonggi-do, Korea) for 96 h in the presence of each EO concentration. For that assay, biofilm formation took place under the optimum (biofilm-maximizing) conditions for each strain (with respect to medium, time and incubation temperature), as these had been previously determined [27]. Thus, *S*. Typhimurium was grown in 1/10 diluted TSB (dTSB) at 20 °C, whereas *L. monocytogenes* was grown in BHI broth at 37 °C. For both bacteria, the growth medium was renewed at 48 h of incubation. At the end of incubation, the accumulated biomasses were quantified through their staining with CV (0.1% *w*/*v*) and subsequently measuring absorbance at 590 nm (A_590nm_). For each bacterial strain, the MBIC of the EO was determined as its lowest concentration that completely inhibited biofilm formation (biomass accumulated did not significantly differ from the negative control). Each experiment was repeated three times starting from independent bacterial cultures.

### 2.5. Determination of Minimum Biofilm Eradication Concentrations (MBECs)

The MBEC of the EO against the already 96 h preformed biofilms of each bacterial strain was determined as previously described [27]. In brief, five different concentrations were tested for the EO which were all prepared in sterile distilled water (dH_2_O) by successive two-fold dilutions and ranged from 6.25 to 0.39% *v*/*v*. Each EO disinfection solution was left to act against the biofilm cells for 15 min at room temperature. For each disinfection treatment, the survivors were enumerated by plate counting on TSA following their removal from surfaces through scratching (with a plastic pipette tip). For each bacterial strain, the MBEC of the EO was determined as its lowest concentration leading to at least six log reductions of the biofilm population compared to that of the negative control (dH_2_O also containing 10% *v*/*v* ethanol). Each experiment was repeated three times starting from independent bacterial cultures.

### 2.6. Chemical Analysis of EO (GC-MS)

Oregano EO was diluted in pure hexane (1:9) and the mixture was then chemically characterized by GC-MS analysis on a 6890N system (Agilent Technologies, Santa Clara, CA, USA) equipped with an HP-5MS column (30 m, 0.25 mm inner diameter, 0.25 μm film thickness) (Agilent Technologies) and coupled with a 5973Network mass detector (Agilent Technologies), as previously described [27].

### 2.7. Sublethal Exposure of Planktonic L. monocytogenes Cells to EO and RNA Extraction

*L. monocytogenes* bacteria from the final 18 h working culture were used to inoculate 10 mL of sterile BHI broth at 1:100 dilution (so as to have an initial concentration of ca. 10^7^ CFU/mL). The new culture was incubated at 37 °C for 6 h and its cells were then collected by centrifugation (5000× *g* for 10 min at 4 °C) and the obtained pellet was subsequently suspended in 4 mL of quarter-strength Ringer’s solution (Lab M). That freshly saline cellular suspension was aliquoted in two Eppendorf^®^ tubes (2 mL in each one) and centrifuged (5000× *g* for 10 min at 4 °C). One of the two bacterial pellets was then suspended in 1 mL of the EO solution (0.063% *v*/*v*; equal to the MBIC), while the second pellet was suspended in 1 mL of dH_2_O to be used as the untreated control sample. The dH_2_O of the control sample also contained absolute ethanol at 0.56% *v*/*v*. This latter was the concentration that existed in the EO solution (and was used to disolve the oil). Both samples were incubated in a heating dry block for 3 h at 37 °C (sublethal EO exposure) and were then immediately centrifuged (5000× *g* for 10 min at 4 °C). Supernatants were discarded and each pellet was washed with dH_2_O through an additional centrifugation step (5000× *g* for 10 min at 4 °C) to remove any EO residues. Washed pellets were then suspended in 1 mL of RNA*later*™ Stabilization Solution (AM7021, Invitrogen™, Thermo Fisher Scientific, Carlsbad, CA, USA) and incubated overnight at 4 °C. Next day, RNA*later*™ Solution was removed by centrifugation (5000× *g* for 10 min at 4 °C) and each bacterial pellet was then resuspended in 100 μL of TE buffer (10 mM Tris-HCl, 1 mM EDTA; pH 8) also containing 2.5 mg/mL lysozyme (native, chicken egg white, 4403, Calbiochem^®^, Merck KGaA) and incubated at 37 °C for 10 min. Following this enzymatic digest of the bacterial cell walls, the resulting homogenates were directly used for RNA extraction using the NucleoSpin^®^ RNA Mini Kit (740955, MACHEREY-NAGEL GmbH & Co., KG, Düren, Germany).

### 2.8. cDNA Synthesis and qPCR

The cDNA synthesis was executed starting from 500 ng of each RNA sample (i.e., exposed and not exposed to the EO) using PrimeScript™ RT reagent Kit (RR037A, Takara Bio Inc., Shiga, Japan), according to the manufacturer’s instructions. Each cDNA template was then used as substrate in qPCR prepared using the FastGene^®^ IC Green 2 × qPCR Universal Mix (LS4005, NIPPON Genetics EUROPE GmbH). Four virulence genes of *L. monocytogenes* were selected (*hly*, *inlA*, *inlB* and *prfA*) for this analysis, which was done as previously described [28]. Two reference (internal control) genes (*tuf*, *gap*) were included in each assay for the normalization of the qPCR data. Each experiment was repeated three times starting from independent bacterial cultures and each reaction was performed in triplicate.

### 2.9. Statistics

Biofilm plate counts (CFU/cm^2^) were transformed to logarithms before means and standard deviations were computed. The derived data on biofilm biomasses (A_590nm_), planktonic absorbances (A_600nm_), and biofilm populations (log_10_ CFU/cm^2^) were all submitted to analyses of variance (ANOVA), followed by Tukey’s multiple range post hoc honestly significant difference (HSD) tests for mean comparison, using the statistical software STATISTICA^®^ (StatSoft Inc., Tulsa, OK, USA). In ANOVA, the dependent variables were the biofilm biomasses (A_590nm_), planktonic absorbances (A_600nm_) and biofilm populations (log_10_ CFU/cm^2^), while the categorical factor was always the treatment (EO concentration). Regarding gene expression, the data derived from a total of 108 reactions were analyzed. These were the result of six different cDNA samples (i.e., one bacterial strain × two treatments (exposed and not exposed to the EO) × three biological repetitions) × six genes (four targets plus two references)/sample × three technical replicates/gene. For each bacterial strain and target gene, unpaired two-tailed Student’s *t*-tests were applied to check for any significant difference in gene expression (expressed as log_2_(fold difference)) between the two treatments. These tests were performed using the relevant function of Excel^®^ module of the Microsoft^®^ Office 365 suite (Redmond, WA, USA). For all statistical analyses, significant differences were reported at a *p* level of <0.05.

## 3. Results and Discussion

### 3.1. Antimicrobial Actions of O. vulgare EO against Planktonic and Biofilm Cells

The minimum inhibitory and minimum bactericidal concentrations (MICs, MBCs) of *O. vulgare* EO against the planktonic cells of *S.* Typhimurium and *L. monocytogenes* were initially determined. The MICs were found equal to 0.06 and 0.03% *v*/*v* against *S.* Typhimurium and *L. monocytogenes*, respectively (Table 1; equal to 0.55 and 0.27 mg/mL, respectively). No differences between the MICs and MBCs were observed, indicating the strong bactericidal action of the EO.

Oregano EO, such as many other EOs, may present multiple modes of action against microbial cells. The reader is referred to specialized reviews on the mode of actions of EOs for more info [1,2]. Many other studies have in the past analyzed the antimicrobial action of *O. vulgare* EOs against planktonic bacteria. For instance, Mazzarrino et al. (2015) determined the MICs of Italian *O. vulgare* EO against *Salmonella* spp. and *L. monocytogenes* and found values ranging from 0.6 to 1.2 μL/mL [29]. Assiri et al. (2016) determined the minimal lethal concentration (MLC) of *O. vulgare* EO from Saudi Arabia against *S.* Enteritidis and *L. monocytogenes* and found values equal to 0.16 and 0.32 mg/mL [30]. Elansary et al. (2108) found MIC and MBC of Egyptian *O. vulgare* EO against *L. monocytogenes* equal to 0.40 and 0.83 mg/mL, respectively [31]. Barbosa et al. (2020) found maximum sublethal concentration (MSC) of Brazilian *O. vulgare* EO against *S.* Enteritidis equal to 0.13 mg/mL [32]. Torabian Kakhki et al. (2020) found MIC and MBC of Iranian *O. vulgare* EO against *L. monocytogenes* equal to 1.28 and 2.56 mg/mL, respectively [33]. Solarte et al. (2020) determined the MIC of Spanish *O. vulgare* EO against one reference *S.* Typhimurium strain and two porcine isolates equal to 0.31 mg/mL [34]. It is clear that the EO from the Greek organically cultured oregano plants tested in the current study presents strong antimicrobial action that is similar or even superior to that previously described for *O. vulgare* EOs from some other countries.

**Figure 1:**
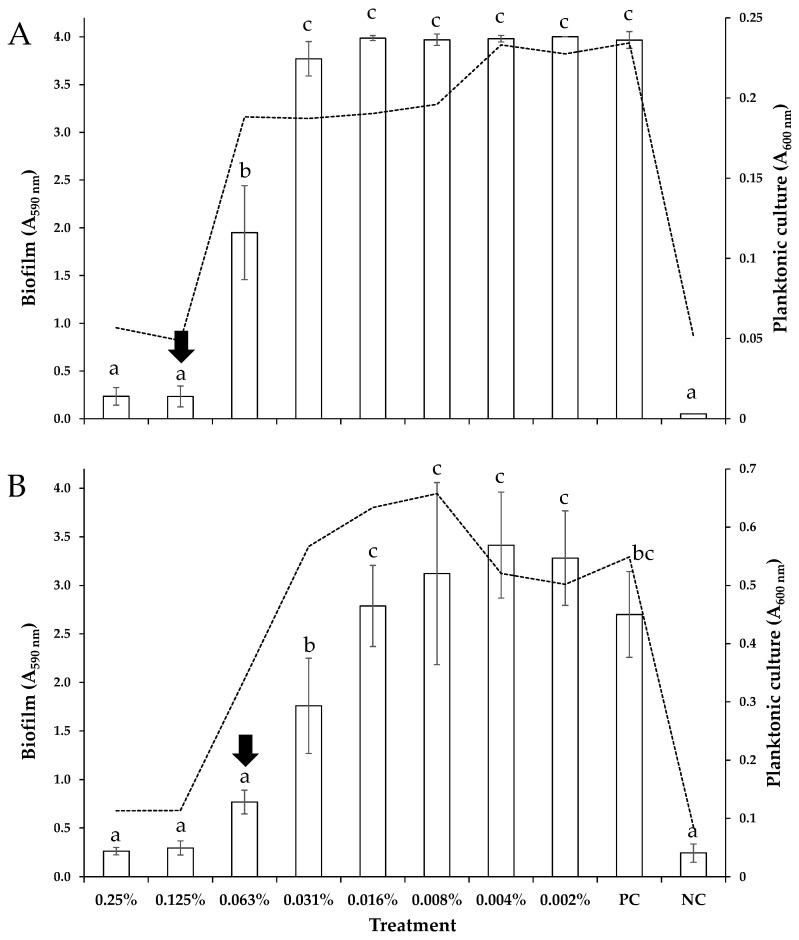
Biofilm formation (A_590nm_) by *S*. Typhimurium (**A**) and *L. monocytogenes* (**B**) strains on the PS surface of the 96-well microtiter plates, in the presence of eight different *O. vulgare* EO concentrations (two-fold dilutions ranging from 0.25 to 0.002% *v*/*v*). The bars represent the mean values ± standard deviations. The accumulated biofilm biomasses of the positive (PC) and negative control (NC) are also shown. For each bacterial strain, the MBIC of the EO (resulting in the total inhibition of biofilm formation) is indicated by the vertical arrow. The absorbances of planktonic suspensions (A_600nm_) found in the same wells at the time of sampling (96 h) are also shown for each treatment (dotted curved lines). The bars of standard deviations of the planktonic means were omitted for clarity. In each graph (bacterial strain), mean biofilm values followed by different superscript letters (abc) differ significantly (*p* < 0.05).

Following the calculation of the MICs and MBCs of the *O. vulgare* EO against the planktonic *S.* Typhimurium and *L. monocytogenes* bacteria, we determined its minimum biofilm inhibitory concentrations (MBICs) against the biofilm growth of those bacteria. This is because both pathogens are known to form robust biofilms on various surfaces (both food-contact and non-food-contact ones) that display increased tolerance to antimicrobial agents and other stresses [35,36]. The protocol we followed to form biofilms had been previously optimized to maximize the sessile growth by each of these two bacterial strains [27]. However, and despite that optimization, the determined MBICs were still quite small and equal to 0.13 and 0.06% *v*/*v* against *S.* Typhimurium and *L. monocytogenes*, respectively (Table 1). This indicates the ability of the *O. vulgare* EO to efficiently inhibit biofilm formation by the tested pathogens at very low concentrations and thus its high antibiofilm potential. Figure 1 presents the biofilm biomasses (A_590nm_) accumulated on the PS surface for each one of the eight tested EO concentrations (0.25–0.002% *v*/*v*). The densities of the surrounding planktonic suspensions (A_600nm_) found in the same wells at the time of sampling (96 h) are also shown for each treatment (as dotted curved lines). It is worth noting that in the case of *L. monocytogenes*, the application of the EO at its MBIC (0.06% *v*/*v*) resulted in the total inhibition of biofilm formation, whereas the surrounding planktonic cells were still able to multiply, although to a lesser extent compared to the positive control (Figure 1B). This is quite interesting since it probably indicates that the observed inhibition of biofilm formation should not be a sole result of the reduced growth rate of the bacteria (before and after their attachment to the surfaces), but it could also be associated with the inhibition of some other biofilm-specific mechanisms, such as intercellular interactions and aggregation, motility, production of extracellular polymeric substances (EPS), altered gene expression etc. It is indeed known that various phytochemicals and plant extracts may display antivirulence abilities and anti-quorum sensing behavior upon their application at sub-MICs [37,38]. Such targeted antibiofilm action at sub-MICs is believed to limit the possibilities of the bacteria to develop resistance [39].

**Figure 2:**
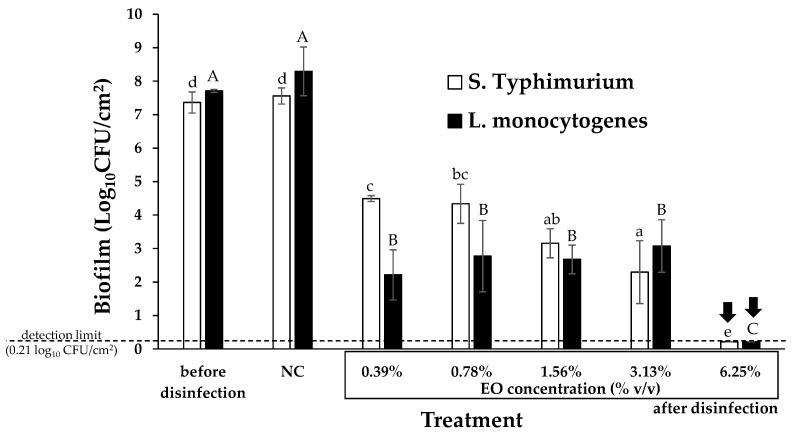
Biofilm populations (log_10_ CFU/cm^2^) found in the wells of the 96-well microtiter plates for the two bacterial strains (*S*. Typhimurium and *L. monocytogenes*) before and after disinfection with the *O. vulgare* EO. For each bacterial strain, the EO was tested in five different concentrations, two-fold dilutions ranging from 0.39 to 6.25% *v*/*v*. The biofilm population of the negative disinfection control (NC; sterile distilled water also containing 10% *v*/*v* ethanol) is also shown. The bars represent the mean values ± standard deviations. For each bacterial strain separately, mean values followed by different superscript letters differ significantly (*p* < 0.05). For each bacterial strain, the MBEC of the EO (resulting in more than six log reductions of its biofilm population with respect to that of the NC) is indicated with the vertical arrow. The detection limit of the plate counting method (0.21 log_10_CFU/cm^2^) is indicated with the horizontal dashed line.

Besides the determination of the MBICs, we also determined the Minimum Biofilm Eradication Concentrations (MBECs) of *O. vulgare* EO against the 96 h preformed biofilms of each target pathogen. Results revealed that the oil needed to be applied at 6.25% *v*/*v* to destroy the biofilm communities of both pathogens (Figure 2). Undoubtedly, this is a quite high concentration that clearly denotes the great recalcitrance of biofilm cells. This is however not surprising since it is well known that the inclusion of bacteria in biofilm structures can greatly increase their tolerance and resistance to biocides, including known antibiotics [40,41]. Thus, in the current experimental setup, *O. vulgare* EO needed to be applied more than one hundred times more than its MBC to kill *S*. Typhimurium biofilm cells, while in the case of *L. monocytogenes* biofilm cells, the required increase in concentration was even more spectacular (more than two hundred times more than its MBC) (Table 1). Another probably interesting observation is that a dose-response behavior does not seem to exist in the case of the killing of biofilm cells (Figure 2). This is more evident in the case of *L. monocytogenes* where the application of *O. vulgare* EO at 0.39% *v*/*v* provoked a ca. five-log reduction in the number of biofilm cells, whereas the further ten-fold increase of the EO concentration to 3.13% *v*/*v* did not further improve its killing action. The reasons lying behind this last observation are not known but we speculate that these may be somehow related to the presence of EPS and/or the complex biofilm tertiary structure [42,43]. A similar behavior has been previously observed in another similar older study of our group where an increase in the concentrations of sage and spearmint EOs from 5% to 15% *v*/*v* did not improve their efficiency against *Staphylococcus aureus* biofilm bacteria [44].

A few other studies have been published in the previous years testing the antibiofilm efficiency of *O. vulgare* EO against biofilm formation and/or preformed biofilms of Salmonella spp. or *L. monocytogenes*. In such a study, Čabarkapa et al. (2019) tested the effectiveness of *O. vulgare* EO against planktonic and biofilm cells of two strains of *S.* Enteritidis [21]. In that study, *O. vulgare* EO demonstrated (as expected) a significant antimicrobial action against the planktonic cells (MIC/MBC = 0.16/0.31 μL/mL). Supplementation of EO at concentration 0.25xMIC caused 50% inhibition of biofilm formation for both the tested strains, while its supplementation at 2xMIC resulted in 90% inhibition of their biofilm formation. Contrary to our results, the study on the effectiveness of EO on eradication of preformed 48 h-old biofilms indicated that biofilm reduction occurred in a dose-dependent manner over time (the time of exposure ranged from 15 to 60 min). In another study, Di Vito et al. (2020) compared the in vitro antibiofilm effectiveness of Italian *O. vulgare* EO against 29 strains of *Salmonella* spp. isolated from poultry and pig farms belonging to two serotypes (*S.* Typhimurium and *S.* Infantis) [45]. *O. vulgare* EO was active on the disaggregation of mature biofilms of both serotypes, but no inhibiting activity was exerted on biofilm formation. Soni et al. (2013) evaluated EOs of thyme and oregano and their antimicrobial phenolic constituent carvacrol for their ability to inhibit biofilm formation and inactivate preformed *Salmonella* biofilms [46]. The presence of nonbiocidal concentrations of thyme oil, oregano oil and phenolic carvacrol at 0.006 to 0.012% *v*/*v* suppressed *Salmonella* spp. biofilm formation two- to four-fold, but could not completely eliminate biofilm formation. Treatment of 1-day-old biofilms with thyme oil, oregano oil or carvacrol at 0.05 or 0.1% *v*/*v* significantly reduced the amount of preformed biofilms. In a recent study, Vidaković Knežević et al. (2023) determined the antibiofilm activity of *O. vulgare* EO from Serbia against five *L. monocytogenes* strains [47]. The MIC values of oregano EO ranged from 0.09 to 0.72 μL/mL. The exposure of 48 h-old *L. monocytogenes* biofilms on PS to the oregano EO at its MBC (2xMIC) for 48 h reduced *L. monocytogenes* biofilm biomasses in the range of 42.9% to 78.6%. Desai et al. (2012) tested oregano EO for its ability to eliminate *L. monocytogenes* biofilms on PS and stainless steel (SS) surfaces [48]. For 1-day old biofilms of mixed *L. monocytogenes* strains produced at 22 °C on PS microtiter plates, only 0.1% *v*/*v* of EO was needed to eliminate 7 log CFU per well. On the SS coupons, 0.5% *v*/*v* was adequate to completely eliminate 4-day-old biofilms at 7 log CFU per coupon. However, in that older study, the EO was left to act for 24 h against the preformed biofilms, something that probably explains its high efficiency. In our study, we chose a short exposure time (15 min) to try to imitate a short disinfection/biocidal protocol that could be applied in the food industry and/or clinical settings.

### 3.2. Chemical Composition of O. vulgare EO

The GC–MS analysis of the *O. vulgare* EO revealed the presence of 28 compounds representing 98.1% of the total oil (Table 2). The EO was mainly composed of the monoterpenes thymol (31.5%), *p*-cymene (23.7%), *γ*-terpinene (13.9%), and carvacrol (8%). All these compounds are commonly found as main constituents of *O. vulgare* EOs and contribute to their bioactivities [2,3,49]. However, in most other studies, the EOs are mainly composed of carvacrol, whereas the EO analyzed in the current study was mainly composed of thymol. This is not strange since some other studies have also described *O. vulgare* EOs of thymol chemotypes [50,51,52,53,54].

### 3.3. Effect of O. vulgare EO on Virulence Gene Expression of Planktonic L. monocytogenes Cells

**Figure 3:**
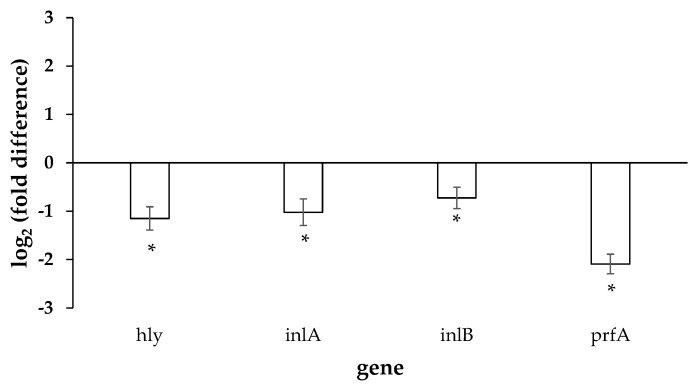
Relative quantification (log_2_(fold differences)) of the expressions of the four target genes (*hly*, *inlA*, *inlB*, and *prfA*) by the planktonic *L. monocytogenes* bacteria following their sub-lethal exposure to the *O. vulgare* EO at its MBIC (0.06% *v*/*v*). Each bar represents the mean ± standard errors (*n* = 9). The statistically significant differences in genes’ expressions between the two treatments (exposed and not exposed to the EO) are indicated with asterisks (*) (*p* < 0.05).

The expression of the four target genes (*hly*, *inlA*, *inlB*, and *prfA*) by the planktonic *L. monocytogenes* bacteria following their sub-lethal exposure to the *O. vulgare* EO is shown in Figure 3. All the studied genes were significantly downregulated following the exposure of the cells to the EO at its MBIC (0.06% *v*/*v*). This is an intriguing result and seems to agree with and is probably related to the inhibition of biofilm formation that was noticed for that EO concentration (Figure 1B). Interestingly, in a previous study of our group, all these four genes were found to be significantly overexpressed when the same *L. monocytogenes* strain (AAL 20107) was left to form biofilm on a PS surface [28]. The *hly* gene encodes in *L. monocytogenes* the hemolysin Listeriolysin O (LLO) which is crucial in the manifestation of listeriosis by boosting the cell-to-cell spread and thus successful dissemination of the pathogen during infection [55]. Both *inlA* and *inlB* genes encode in *L. monocytogenes* for important surface proteins that are known to interact with distinct host receptors to cause infection of human cells and crossing of the intestinal, blood–brain or placental barriers [56]. *PrfA* encodes in *L. monocytogenes* for the master regulator of virulence that controls the expression of multiple virulence factors that altogether facilitate the transition from extra- to intracellular environments [57]. Rather in agreement with our results, some other previous studies have shown the involvement of all these four genes in attachment, sessile growth, and biofilm formation by *L. monocytogenes* [58,59,60,61]. In addition, the reduced expression of *hly* and *prfA* genes in *L. monocytogenes* following exposure to some other EOs has been shown in some other previous studies as well [62,63,64].

## 4. Conclusions

*Origanum vulgare* subsp. *hirtum* EO, extracted from organically cultivated plants on the Greek island of Lemnos was found to present strong antimicrobial and antibiofilm actions against two important foodborne pathogenic bacteria (*S*. Typhimurium and *L. monocytogenes*). Its application at 0.13% *v*/*v* and 0.06% *v/v* was sufficient to inhibit biofilm formation by *S*. Typhimurium and *L. monocytogenes*, respectively, whereas this needed to be applied at 6.25% *v*/*v* (for 15 min) to eradicate the preformed biofilms of the same species. The EO was mainly composed of thymol, *p*-cymene, *γ*-terpinene and carvacrol. The sub-lethal exposure *of L. monocytogenes* planktonic cells to the EO at its MBIC (0.06% *v*/*v*) resulted in the significant downregulation of four important virulence genes (*hly*, *inlA*, *inlB*, and *prfA*). To sum, the results obtained advocate for the further exploitation of the antimicrobial action of oregano EO in food and health applications. Future studies could be conducted on some such applications. In addition, the specific mode of action of this EO against microbial cells could be tested by applying either the crude extract or some of its individual components.

## Figures and Tables

**Table 1:** Antibacterial (MIC and MBC values) and antibiofilm (MBIC and MBEC values) actions of *O. vulgare* EO against *S*. Typhimurium and *L. monocytogenes* strains expressed as either EO percentages by volume (% *v*/*v*) or μL/mL.

Bacterial Species	Antibacterial Action				Antibiofilm Action			
	MIC ^1^		MBC ^2^		MBIC ^3^		MBEC ^4^	
	% (*v*/*v*)	μL/mL	% (*v*/*v*)	μL/mL	% (*v*/*v*)	μL/mL	% (*v*/*v*)	μL/mL
*S.* Typhimurium	0.06	0.6	0.06	0.6	0.13	1.3	6.25 (=104.2 × MΒC)	62.5
*L. monocytogenes*	0.03	0.3	0.03	0.3	0.06	0.6	6.25 (=208.4 × MΒC)	62.5

^1^ Minimum Inhibitory Concentration; ^2^ Minimum Bactericidal Concentration; ^3^ Minimum Biofilm Inhibitory Concentration; ^4^ Minimum Biofilm Eradication Concentration.

**Table 2:** Chemical composition of *O. vulgare* EO, as characterized by GC-MS analysis.

Compounds Detected	LRΙ	% Area
Methyl-cyclopentane	<700	0.8
Methyl α-methylbutanoate	781	0.1
*α*-Thujene	930	2.6
*α*-Pinene	936	2.3
Camphene	949	0.6
*β*-Pinene	977	0.4
1-Octen-3-ol	997	0.2
*β*-Myrcene	999	3.4
*α*-Phellandrene	1008	0.7
3-Carene	1012	0.2
*α*-Terpinene (1-methyl-4-(1-methylethyl)-1,3-cyclohexadiene)	1020	4.4
*p*-Cymene (1-methyl-4-(1-methylethyl)-benzene)	1034	23.7
Sylvestrene ((R)-1-methyl-5-(1-methylethenyl)-cyclohexene)	1035	2.4
*p*-Cymenene (1-methyl-4-(1-methylethenyl)-benzene)	1044	0.1
*β*-cis-Ocimene	1062	0.1
*γ*-Terpinene (1-methyl-4-(1-methylethyl)-1,4-cyclohexadiene)	1070	13.9
*α*-Terpinolene (1-methyl-4-(1-methylethylidene)-cyclohexene)	1095	0.3
Linalool (3,7-dimethyl-1,6-octadien-3-ol)	1123	0.1
Borneol	1183	0.4
Terpinen-4-ol (4-methyl-1-(1-methylethyl)-3-cyclohexen-1-ol)	1191	0.7
*α*-Terpineol (α,α-4-trimethyl-3-cyclohexene-1-methanol)	1209	0.1
1-methoxy-4-methyl-2-(1-methylethyl)-benzene	1262	0.8
Thymol	1341	31.5
Carvacrol (2-methyl-5-(1-methylethyl)-phenol)	1346	8.0
Caryophyllene	1439	0.8
*α*-Caryophyllene	1475	0.1
*β*-Bisabolene ((S)-1-methyl-4-(5-methyl-1-methylene-4-hexenyl)-cyclohexene)	1532	0.1
Caryophyllene oxide	1610	0.1
Total		98.1

LRI: Linear retention index.

## Data Availability

The data presented in this study are available upon reasonable request form the corresponding author.
